# Effect of holding equine oocytes in meiosis inhibitor-free medium before in vitro maturation and of holding temperature on meiotic suppression and mitochondrial energy/redox potential

**DOI:** 10.1186/1477-7827-12-99

**Published:** 2014-10-11

**Authors:** Nicola A Martino, Maria E Dell’Aquila, Manuel Filioli Uranio, Lucia Rutigliano, Michele Nicassio, Giovanni M Lacalandra, Katrin Hinrichs

**Affiliations:** Veterinary Clinics and Animal Productions Unit–Dipartimento dell’Emergenza e Trapianti D’Organo (DETO), Università di Bari Aldo Moro, Str. Prov. Casamassima Km 3°, Valenzano, 70010 Bari Italy; Departments of Veterinary Physiology & Pharmacology and Large Animal Clinical Sciences, TAMU 4466, Texas A&M University, College Station, TX 77843-4466 USA

**Keywords:** Pre-maturational oocyte holding, Nuclear chromatin configuration, Germinal vesicle, Mitochondria, Reactive oxygen species

## Abstract

**Background:**

Evaluation of mitochondrial function offers an alternative to evaluate embryo development for assessment of oocyte viability, but little information is available on the relationship between mitochondrial and chromatin status in equine oocytes. We evaluated these parameters in immature equine oocytes either fixed immediately (IMM) or held overnight in an Earle’s/Hank’s’ M199-based medium in the absence of meiotic inhibitors (EH treatment), and in mature oocytes. We hypothesized that EH holding may affect mitochondrial function and that holding temperature may affect the efficiency of meiotic suppression.

**Methods:**

Experiment 1 - Equine oocytes processed immediately or held in EH at uncontrolled temperature (22 to 27°C) were evaluated for initial chromatin configuration, in vitro maturation (IVM) rates and mitochondrial energy/redox potential. Experiment 2 - We then investigated the effect of holding temperature (25°C, 30°C, 38°C) on initial chromatin status of held oocytes, and subsequently repeated mitochondrial energy/redox assessment of oocytes held at 25°C vs. immediately-evaluated controls.

**Results:**

EH holding at uncontrolled temperature was associated with advancement of germinal vesicle (GV) chromatin condensation and with meiotic resumption, as well as a lower maturation rate after IVM. Holding did not have a significant effect on mitochondrial distribution within chromatin configurations. Independent of treatment, oocytes having condensed chromatin had a significantly higher proportion of perinuclear/pericortical mitochondrial distribution than did other GV configurations. Holding did not detrimentally affect oocyte energy/redox parameters in viable GV-stage oocytes. There were no significant differences in chromatin configuration between oocytes held at 25°C and controls, whereas holding at higher temperature was associated with meiosis resumption and loss of oocytes having the condensed chromatin GV configuration. Holding at 25°C was not associated with progression of mitochondrial distribution pattern and there were no significant differences in oocyte energy/redox parameters between these oocytes and controls.

**Conclusions:**

Mitochondrial distribution in equine GV-stage oocytes is correlated with chromatin configuration within the GV. Progression of chromatin configuration and mitochondrial status during holding are dependent on temperature. EH holding at 25°C maintains meiotic arrest, viability and mitochondrial potential of equine oocytes. This is the first report on the effects of EH treatment on oocyte mitochondrial energy/redox potential.

## Background

Use of a modified Earle’s/Hank’s M199 (EH medium, [1]) at room temperature has been shown to effectively maintain equine oocytes in meiotic arrest in the absence of meiotic inhibitors. This oocyte holding technique has been utilized in studies on both intracytoplasmic sperm injection (ICSI) and nuclear transfer [2–8], to enable scheduling of in vitro maturation (IVM) and subsequent manipulations. In two studies, in which direct comparisons were performed, overnight EH holding was associated with equine blastocyst developmental rates after IVM and ICSI equivalent to those for controls: 34% for EH holding vs 25% for immediate culture [1]; and 23% for EH holding vs 17% for culture with roscovitine [3]. This holding technique is utilized due to the fact that the maturation duration needed for optimum developmental competence of equine oocytes is about 30 hours [9]. Holding immature oocytes before IVM allows scheduling of the onset of maturation [1] and may also allow overnight transport of oocytes from the field to laboratories that can effectively perform ICSI [6, 8].

Despite the growing use of holding equine immature oocytes for both clinical and research applications, little information is available on the effect of holding conditions on oocyte metabolism, or the effect of EH holding on pregnancy or foaling rates after transfer of blastocysts from these oocytes. It is possible that holding could positively affect cytoplasmic maturation, as previously hypothesized for culture in the presence of meiotic inhibitors [1, 10–13], or, alternatively, that it may have negative consequences for oocyte developmental potential. In addition, the mode by which this method maintains meiotic arrest is currently unknown; from initial findings in our study, we hypothesized that holding temperature affects oocyte meiotic arrest.

The ideal method for determining the effect of holding conditions on oocyte developmental competence is to investigate embryo development, pregnancy and foaling after maturation and fertilization of held oocytes. However, in the horse, this is not only laborious and expensive, but also problematic for most laboratories. Repeatable methods for standard in vitro fertilization (IVF) have still not been developed in the horse, thus ICSI is currently used to fertilize equine oocytes in vitro for both research [4, 9, 14, 15] and clinical applications [6, 16]. Only one laboratory has published multiple reports presenting effective (>15% per injected oocyte) in-vitro production of equine ICSI blastocysts [2, 9, 17–19]; other publications commonly report blastocyst rates per injected oocyte of <10% [20–25]. For these reasons, in vitro production of blastocysts followed by transfer to mares cannot be easily applied as a sensitive test of equine oocyte cytoplasmic competence in most laboratories. Therefore, the identification of alternative objective oocyte evaluation methods is needed.

An alternative method for evaluation of oocyte metabolic function is determination of mitochondrial activity. Mitochondria serve to power oocyte maturation and are crucial for supporting events occurring downstream of sperm penetration [26]. The Mitochondria present in the oocyte at the time of fertilization are responsible for early embryonic development, as Mitochondria replication does not start in the developing embryo until around the time of blastocyst formation [27, 28]. Mitochondria affect intracellular redox potential by regulating the NAD(P)H:NAD(P)^+^ ratio and by producing reactive oxygen species (ROS; [29, 30]). The redox state of the oocyte, in turn, affects the kinetics of Ca^2+^ oscillations at activation [26] and regulates developmentally-important transcription factors [31, 32], chromatin remodeling [33], and embryo and fetal development, even influencing postnatal body weight [34]. Reactive oxygen species play important roles in intracellular signalling, and colocalization of ROS and Mitochondria has been related to cell health in hepatocytes [35] and in oocytes [36, 37]. Thus, the proper localization and activity of Mitochondria within the oocyte have a direct effect on early embryo development.

Patterns of mitochondrial distribution in mature oocytes vary among species [38]. Mitochondrial distribution and activity patterns of horse oocytes during maturation have been previously described [39, 40] and our research group has established a confocal laser scanning microscopy based multiparametric method for assessment of oocyte mitochondrial energy/redox status that provides data consistent with biochemical biomarkers, such as ATP content and scavenger enzyme activity [41].

In the present study, we used multiparametric epifluorescence/confocal microscopy-based analysis to examine the relationship of mitochondrial energy/redox status to GV stage (chromatin configuration within the GV) and meiotic maturation, and to evaluate the effects of EH treatment on these parameters. The effect of temperature on efficiency of meiotic suppression during EH treatment was also evaluated.

## Methods

The study was conducted in Southern Italy (41° North parallel) during four subsequent breeding seasons (2011 to 2014). The study was performed in accordance with the ethical standards laid down in the 1964 Declaration of Helsinki and its subsequent amendments. All the procedures with animals were performed following good veterinary practice for animal welfare according to the Italian law (D.Lgs 116/92).

### Chemicals

All chemicals were purchased from Sigma-Aldrich (Milano, Italy) unless otherwise indicated.

### Collection of cumulus-oocyte complexes

Ovaries from mares of unknown reproductive history were obtained at two local abattoirs within 3 hours of slaughter, transported to the laboratory within 30–45 min after collection in a thermal container set at 30°C and processed for recovery of cumulus-oocyte complexes (COCs). A follicle-scraping procedure on follicles 0.5 to 2.5 cm in diameter was used, as previously described [14]. Only COCs classified as having an intact cumulus investment and intact oocyte plasma membrane [14] were selected for culture.

### Oocyte holding and study groups

Immature non cultured-oocytes were analyzed either immediately (IMM) or after overnight holding (EH). For IMM oocytes, the time interval between follicle scraping and beginning of COC processing for analysis or onset of IVM culture was less than 2 hours. COCs to be evaluated after IVM were placed into IVM medium either immediately (IMM-IVM) or after overnight holding (EH-IVM). After IVM culture, only oocytes that were at the metaphase II (MII) stage, as visualized after staining, were analyzed for energy/redox status.

COCs assigned to the EH treatment were placed in 5 ml of a mixture of 40% Earle’s and 40% Hank’s salts-buffered M199 with 20% fetal calf serum (FCS) in glass vacuum vials (Venoject – Terumo vt-050sp, Madrid, Spain). Fifteen to 50 COCs were put into each vial and the vial was sealed with a cap and paraffin film. The vial was then laid down on its side and covered with aluminium foil to block exposure to room light, and kept at room temperature for 16–18 h. After this holding period, EH COCs were directly evaluated or were placed in IVM culture (EH-IVM).

In Exp. 1, COCs were held at largely uncontrolled room temperature, estimated ranging from 22 to 27°C.

In Exp. 2, the effects of three holding temperatures (25°C, 30°C, and 38°C) on oocyte initial chromatin configuration were compared with those for immediately-fixed (IMM) oocytes. EH COCs were held either under air conditioning set at 25°C (EH-25 group) or in a water bath set at 30°C (EH-30 group) or in a sealed container in a cell culture CO_2_ incubator set at 38.2°C (EH-38 group).

### In vitro maturation

In vitro maturation was performed following the procedure described by Dell’Aquila et al. [14], using TCM-199 with Earle’s salts, buffered with 4.43 mM HEPES and 33.9 mM sodium bicarbonate and supplemented with 0.1 g/L L-glutamine, 2 mM sodium pyruvate, 2.92 mM calcium-L-lactate penthahydrate (Fluka 21175 Serva Feinbiochem GmbH & Co Heidelberg, Germany No. 29760), 50 μg/mL gentamicin, 20% (v/v) FCS, gonadotrophins (10 μg/mL ovine FSH and 20 μg/mL ovine LH) and 1 μg/mL 17β estradiol was used. COCs were cultured for 30 h at 38.2°C under 5% CO_2_ in air.

### Oocyte Mitochondria and ROS staining

COCs were denuded of cumulus cells by pipetting in 0.05% trypsin–0.02% EDTA (ECM0920D; Euroclone, Milan, Italy; immature COCs) or TCM 199 with 20% FCS containing 80 IU hyaluronidase/mL (mature COCs). Degenerating oocytes (having shrunken, misshapen or fragmented cytoplasm) and oocytes not in MII after IVM, as evaluated after staining, were discarded. Oocytes were washed and then incubated for 30 min in PBS with 3% bovine serum albumin (BSA) containing 280 nM MitoTracker Orange CMTM Ros (Molecular Probes M-7510, Oregon, USA) at 38.5°C under 5% CO_2_. Oocytes were then washed and incubated for 15 min in PBS with 0.3% BSA containing 10 μM 2′,7′-dichlorodihydrofluorescein diacetate (DCDHF DA) to detect intracellular ROS [37, 41]. Oocytes were then washed and fixed overnight at 4°C with 2% paraformaldehyde solution in PBS.

### Oocyte nuclear chromatin evaluation

After labeling for Mitochondria and ROS determination, oocytes were stained with 2.5 μg/ml Hoechst 33258 in 3:1 (v/v) glycerol/PBS and kept at 4°C in the dark until observation. Nuclear chromatin status was observed under a Nikon Eclipse 600 fluorescent microscope equipped with a B2A (346 nm excitation/460 nm emission) filter and was classified according to the criteria of Dell’Aquila et al. [42] and Hinrichs et al. [9]. Chromatin configurations of germinal vesicle (GV)-stage oocytes were classified as Fluorescent Nucleus (FN), having fluorescence throughout the nucleus; Fibrillar, having chromatin strands throughout the estimated area of the nucleus; Intermediate, having chromatin strands or irregular masses of chromatin over approximately half the estimated nuclear area; or Condensed Chromatin (CC), having chromatin condensed into one small regular or irregular mass. Oocytes having resumed meiosis were characterized as prometaphase I (PI), having chromatids in a circular array, or metaphase I (MI), or MII with first polar body (PB) extruded. Oocytes in anaphase or telophase were rare and were classified as MII. After IVM, only oocytes classified as MII were evaluated further. Oocytes with fragmented or non-detected chromatin were considered to be abnormal and were discarded.

### Assessment of oocyte mitochondrial distribution pattern

Mitochondrial distribution pattern was evaluated at 630× magnification under oil immersion with a Nikon C1/TE2000-U laser scanning confocal microscope. A helium/neon laser ray at 543 nm and the G-2 A filter (551 nm exposure/576 nm emission) was used to evaluate MitoTracker Orange CMTM Ros fluorescence. An argon ion laser ray at 488 nm and B-2 A filter (495 nm exposure/519 nm emission) was used to evaluate DCF fluorescence. Scanning to allow three-dimensional distribution analysis was conducted utilizing 25 optical series from the top to the bottom of the oocyte, with a step size of 0.45 μm. Criteria for mitochondrial pattern definition were based on previous studies in equine oocytes [39–41]: oocytes were separated into two groups as having a homogeneous/fine pattern of fluorescence due to small mitochondrial aggregates (SA) diffused throughout the cytoplasm, which was considered an indication of cytoplasmic immaturity, or an heterogeneous perinuclear/pericortical (P/P) distribution pattern of larger mitochondrial aggregates, with accumulation around the nucleus and in the periphery, considered to be characteristic of cytoplasmic maturation. Oocytes showing irregular distribution of large mitochondrial clusters were classified as abnormal and were not analyzed further.

### Quantification of Mitotracker Orange CMTM Ros and DCF fluorescence intensity

Fluorescence intensity was measured in each oocyte, at the equatorial plane, with the aid of the EZ-C1 Gold Version 3.70 software platform for Nikon C1 confocal microscope, as described previously [37, 41]. Fluorescence intensity within the programmed scan area was recorded and plotted against the conventional pixel unit scale (0–255). Images were taken under fixed conditions with respect to laser energy, signal detection (gain) and pinhole size.

### Mitochondria-ROS colocalization analysis

Colocalization analysis of Mitochondria and ROS was performed with the EZ-C1 Gold Version 3.70 software. Degree of colocalization was reported as a Pearson’s correlation coefficient quantifying the overlap degree between Mitotracker and DCF fluorescence signals [37, 43].

### Statistical analysis

The proportions of oocytes showing the different chromatin configurations and mitochondrial distribution patterns were compared among groups by Chi-square test with the Yates correction for continuity. The Fisher’s Exact Test was used when a value of <5 was expected in any cell. For confocal quantification analysis of mitochondrial activity, intracellular ROS levels and Mitochondria/ROS colocalization, mean values of MitoTracker CMTM Ros and DCF fluorescence intensity and Pearson’s correlation coefficient were compared by one-way ANOVA followed by Multiple Comparison methods (Dunn’s or Holm-Sidak; Sigma Plot software version 11.0). All comparisons were performed between treated and control groups and among meiotic stages. A level of P <0.05 was considered significant.

## Results

### Experiment 1: effects of EH treatment on initial nuclear chromatin configuration, meiotic competence and mitochondrial energy/redox potential of equine oocytes

**Figure 1:**
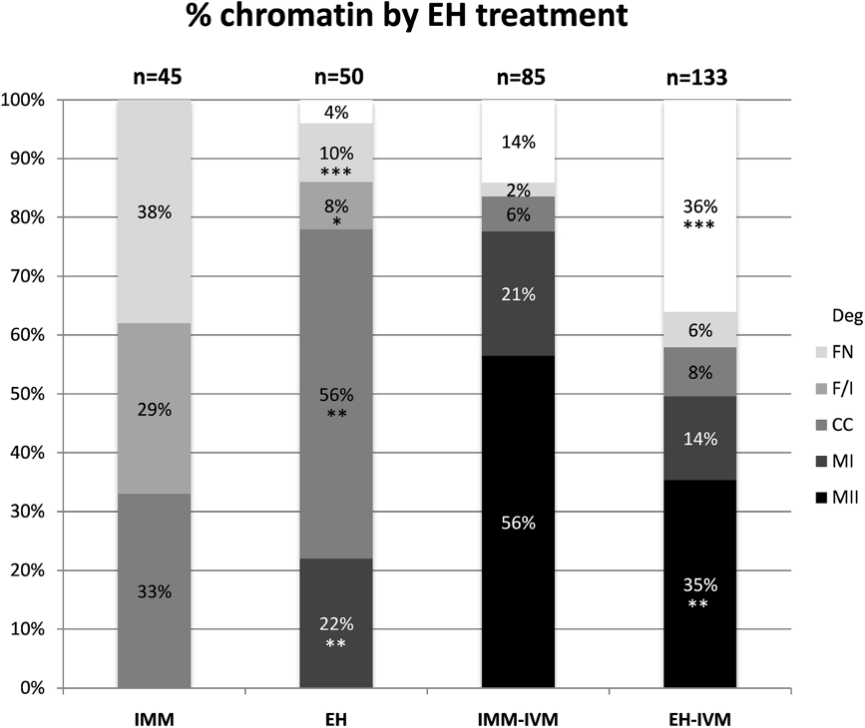
**Effects of holding in EH medium on initial chromatin configuration and meiotic competence of equine oocytes.** Legend for meiotic stages: FN = Homogenous/heterogeneously fluorescent nucleus; F/I = Fibrillar/Intermediate; MI = Metaphase I; MII = Metaphase II, Deg = degenerated chromatin. Legend for treatments: EH = EH treatment (non-cultured); IMM = Immediate fixing (non cultured); EH-IVM = EH treatment and subsequent in vitro maturation; IMM-IVM = Immediate in vitro maturation. Chi Square test with the Yates correction: within each group (Non cultured or cultured for IVM) and for each meiotic stage, EH vs IMM: *P <0.05, **P <0.01 , ***P <0.001; EH-IVM vs IMM-IVM: **P <0.01 , ***P <0.001.

A total of 95 non-cultured oocytes was evaluated for chromatin configuration, 45 in the IMM group and 50 in the EH group, comprising four replicates. EH holding at uncontrolled room temperature significantly affected the chromatin configuration of non-cultured oocytes (Figure 1). EH treatment was associated with a significant decrease in the more juvenile FN (P <0.001) and Fibrillar/Intermediate chromatin configurations (P <0.05), and with a significant increase in oocytes exhibiting the more meiotically competent CC configuration (P <0.01). Notably, 22% of oocytes in the EH treatment had resumed meiosis (prometaphase I and metaphase I), vs 0 in the IMM treatment (P <0.01).

Of these 95 oocytes, 11 were not evaluated further due to degenerated chromatin (n =2) or breakage during processing (n =9). The mitochondrial distribution pattern in the remaining 84 oocytes is presented in Table 1. Thirteen oocytes (9 in the IMM treatment and 4 in the EH treatment) exhibited abnormal mitochondrial distribution; this was highest in IMM oocytes having the FN configuration (44%). There were no significant differences in mitochondrial distribution within chromatin configurations between EH and IMM oocytes (P >0.1), thus, results for oocytes within the IMM and EH treatments were pooled to determine the association of chromatin configuration with mitochondrial distribution. Oocytes in the CC configuration had a significantly higher proportion of the more mature P/P mitochondrial distribution (16/32, 50%) than did oocytes having Fibrillar/Intermediate chromatin (2/15, 13%; P <0.05).

**Table 1:** **Effects of holding in EH medium on mitochondrial distribution pattern of equine oocytes examined before or after IVM**

Treatment	Nuclear chromatin configuration	n. of analyzed oocytes	Small mitochondrial aggregates (SA) n. (%)	Perinuclear/pericortical (P/P) n. (%)	Abnormal n. (%)
**EH**	FN	4	4 (100)	0 (0)	0 (0)
	F/I	2	2 (100)	0 (0)	0 (0)
	CC	24	9 (38)	11 (46)	4 (17)
	PI/MI	11	7 (64)	4 (36)	0 (0)
**IMM**	FN	16	6 (38)	3 (19)	7 (44)
	I	13	11 (84)	2 (15)	0 (0)
	CC	14	7 (50)	5 (36)	2 (14)
	PI/MI	0	0 (0)	0 (0)	0 (0)
**EH-IVM**	MII	34	9 (26)^a^	23 (68)^a^	2 (6)
**IMM-IVM**	MII	46	25 (55)^b^	18 (39)^b^	3 (6)

Legend: Homogenous/heterogeneously fluorescent nucleus (*FN*); Fibrillar/Intermediate (*F/I*); Condensed chromatin (*CC*); Prometaphase I/Metaphase I (*PI/MI*); Metaphase II (*MII*).

Chi square test with the Yates correction: between treatments and chromatin configurations: ^a vs. b^ (P <0.05).

Quantitative data of fluorescence intensities for Mitotracker Orange CMTM Ros and DCF for 71 non-cultured oocytes are reported in Table 2; 13 oocytes which had an abnormal mitochondrial distribution pattern were excluded from quantitative analysis. There was no effect of EH treatment on mitochondrial activity, intracellular ROS levels, or Mitochondria/ROS colocalization either when grouped as total non-cultured oocytes (including oocytes at the PI/MI stage) or when grouped as total GV-stage oocytes. In oocytes having the CC chromatin configuration, EH treatment did not affect quantitative energy/redox parameters. For oocytes having the FN chromatin configuration, EH treatment was associated with significantly lower mitochondrial activity (P <0.05) and Mitochondria/ROS colocalization (P <0.05) and a tendency for lower DCF fluorescence (P =0.08).

**Table 2:** **Effects of holding in EH medium on energy/redox parameters of equine oocytes examined before or after IVM**

Treatment	Nuclear chromatin configuration	n. of analyzed oocytes	Mitochondrial activity	Intracellular ROS levels	Mitochondria/ROS colocalization
EH	FN	4	387.42 ± 347.10^a^	661.20 ± 404.12	0.40 ± 0.13^a^
IMM		9	1248.63 ± 657.93^b,*^	1330.73 ± 651.51	0.64 ± 0.18^b^
EH	F/I	2	§	§	§
IMM		13	273.23 ± 142.95	709.72 ± 423.16	0.56 ± 0.23
EH	CC	20	545.15 ± 283.98	829.89 ± 336.91	0.63 ± 0.16
IMM		12	509.19 ± 242.27**	882.61 ± 486.82	0.67 ± 0.14
EH	PI/MI	11	653.80 ± 191.53	946.64 ± 561.87	0.71 ± 0.11
IMM		0	§	§	§
EH -IVM	POST-IVM MII	32	1112.45 ± 494.10	1434.54 ± 686.00	0.76 ± 0.14
IMM-IVM		43	1372.64 ± 481.34	1982.04 ± 762.74	0.84 ± 0.13

Legend: Homogenous/heterogeneously fluorescent nucleus (*FN*); Fibrillar/Intermediate (*F/I*); Condensed chromatin (*CC*); Prometaphase I/Metaphase I (*PI/MI*); Metaphase II (*MII*). Mitochondrial activity and intracellular ROS levels are presented as MitoTracker and DCF fluorescence intensities expressed in Arbitrary Densitometric Units (*ADU*). Mitochondria/ROS colocalization is expressed as Pearson’s correlation coefficient. One-way ANOVA followed by Multiple Comparison Dunn’s method: ^a,b^ P <0.05; *, **P =0.004; § values for categories with fewer than 3 oocytes are not shown.

Overall, 218 in vitro-cultured oocytes (six replicates for each treatment) were evaluated. Oocyte maturation rate in the IMM-IVM group was significantly higher than that for the EH-IVM group (48/85, 56.5% vs 47/133, 35.3%, respectively; P <0.01, Figure 1). Forty-six IMM MII and 34 EH MII oocytes underwent confocal analysis of energy/oxidative status; the remaining 2 IMM MII and 13 EH MII oocytes were not analysed, as they were damaged during handling. The proportion of oocytes showing the heterogeneous P/P distribution pattern was significantly higher (P <0.05) in the EH-IVM than in the IMM-IVM treatments (Table 1). Fluorescence intensities of Mitotracker Orange CMTM Ros and DCF evaluated in MII oocytes after IVM are reported in Table 2; 5 oocytes which had an abnormal mitochondrial distribution pattern were excluded from quantification analysis. Measured energy status, intracellular ROS levels and Mitochondria/ROS colocalization did not differ between EH-IVM and IMM-IVM MII oocytes. In comparing energy/redox data between non-cultured and MII oocytes, MII oocytes, independent of treatment, showed significantly higher values for all energy/redox parameters than did total non-cultured oocytes (P <0.05; Table 2). In addition, EH-IVM MII oocytes showed significantly higher rates of P/P mitochondrial pattern than did EH (non-cultured immature) oocytes (23/34, 67.6% vs 15/41, 45.3%; P <0.05, Table 1).

### Experiment 2: effect of holding temperature on initial chromatin configuration, meiotic competence and energy/oxidative potential status of equine oocytes

Because the results obtained in Exp. 1, conducted under variable temperature, indicated that EH treatment failed to completely suppress meiosis, in contrast to previous reports [1], a second experiment was conducted to determine whether the holding temperature could affect the ability of EH treatment to suppress meiosis.

**Figure 2:**
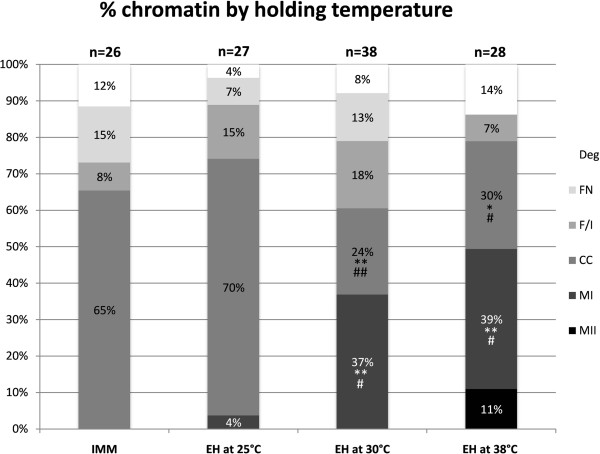
**Effects of holding temperature on initial chromatin configuration of immature non-cultured equine oocytes.** Legend for meiotic stages as in Figure 1. Chi Square test with the Yates correction, for each meiotic stage: i) IMM vs EH at 25°C: not significant; IMM vs EH at 30°C: **P <0.01; IMM vs EH at 38°C: *P <0.05, **P <0.01; ii) 25°C vs 30°C :#P <0.01; ##P <0.001; 25°C vs 38°C: #P <0.01; iii) EH at 30°C vs EH at 38°C: not significant.

One hundred and nineteen oocytes were evaluated in five replicates. Holding temperature significantly affected oocyte nuclear chromatin configuration (Figure 2). When oocytes were held in EH at 25°C, no differences in chromatin configuration were observed in comparison with the IMM group; however, holding at higher temperatures (30°C and 38°C) was associated with significant increases in oocytes resuming meiosis (metaphase I and metaphase II) compared with oocytes in the IMM or 25°C EH held groups.

**Figure 3:**
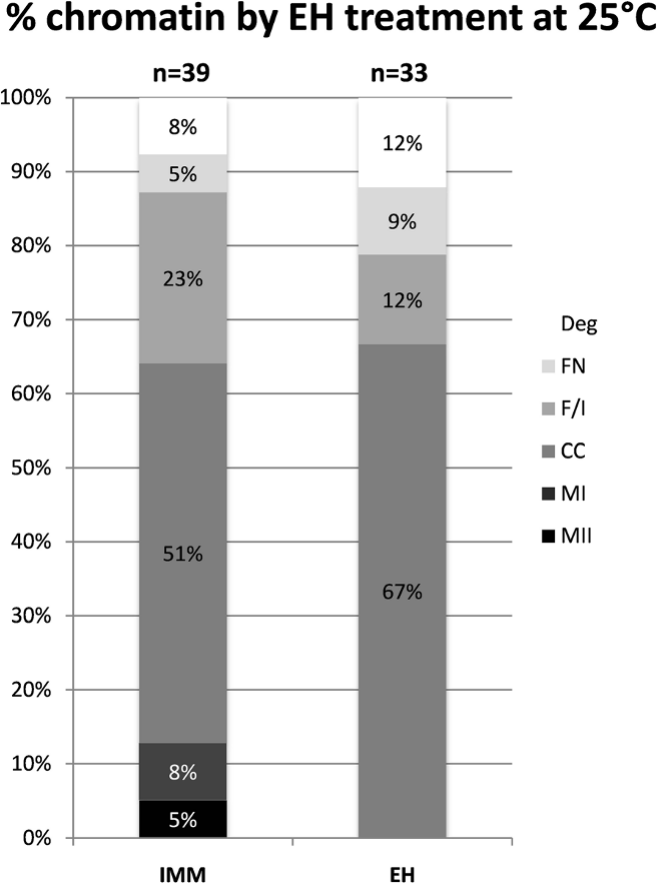
**Effects of holding in EH medium at controlled room temperature (25°C) on initial chromatin configuration of equine oocytes.** Legend for meiotic stages as in Figures 1 and 2. Chi Square test with the Yates correction: for each meiotic stage, EH vs IMM: not significant.

Subsequently, in order to determine whether holding at 25°C maintained Mitochondria/ROS equivalent to that for IMM oocytes, oocytes held in EH at controlled room temperature (25°C) were analysed for initial chromatin configuration, meiotic competence and mitochondrial energy/redox potential. A total of 72 non-cultured oocytes was evaluated in two replicates for chromatin configuration, 39 in the IMM group and 33 in the EH group. EH treatment performed at 25°C did not affect oocyte initial chromatin configuration (Figure 3), as no differences were observed in the rates of oocytes of any chromatin configuration between EH and IMM groups. Of these 72 oocytes, 7 were not evaluated further due to degenerated chromatin (n =4 in the EH group and n =3 in the IMM group). The mitochondrial distribution pattern in the remaining 65 oocytes is presented in Table 3. Two oocytes in the EH treatment exhibited abnormal mitochondrial distribution and were excluded from further analysis. There were no significant differences in mitochondrial distribution within chromatin configurations between EH and IMM oocytes (P >0.1). In the IMM group, three oocytes were found at the metaphase I stage and two oocytes were in metaphase II with the second polar body extruded. All these oocytes exhibited the P/P mitochondrial distribution pattern.

**Table 3:** **Effects of holding in EH medium at controlled room temperature (25°C) on mitochondrial distribution pattern of equine oocytes examined before or after IVM**

Treatment	Nuclear chromatin configuration	n. of analyzed oocytes	Small mitochondrial aggregates (SA) n. (%)	Perinuclear/pericortical (P/P) n. (%)	Abnormal n. (%)
**EH**	FN	3	3 (100)	0 (0)	0 (0)
	F/I	4	4 (100)	0 (0)	0 (0)
	CC	22	12 (55)	8 (36)	2 (9)
	PI/MI	0	0 (0)	0 (0)	0 (0)
	MII	0	0 (0)	0 (0)	0 (0)
**IMM**	FN	2	2 (100)	0 (0)	0 (0)
	F/I	9	4 (44)	5 (56)	0 (0)
	CC	20	6 (30)	14 (70)	0 (0)
	PI/MI	3	0 (0)	3 (100)	0 (0)
	MII	2	0 (0)	2 (100)	0 (0)

Legend: Homogenous/heterogeneously fluorescent nucleus (*FN*); Fibrillar/Intermediate (*F/I*); Condensed chromatin (*CC*); Prometaphase I/Metaphase I (*PI/MI*); Metaphase II (*MII*).

Chi square test with the Yates correction: between treatments and chromatin configurations: NS.

**Figure 4:**
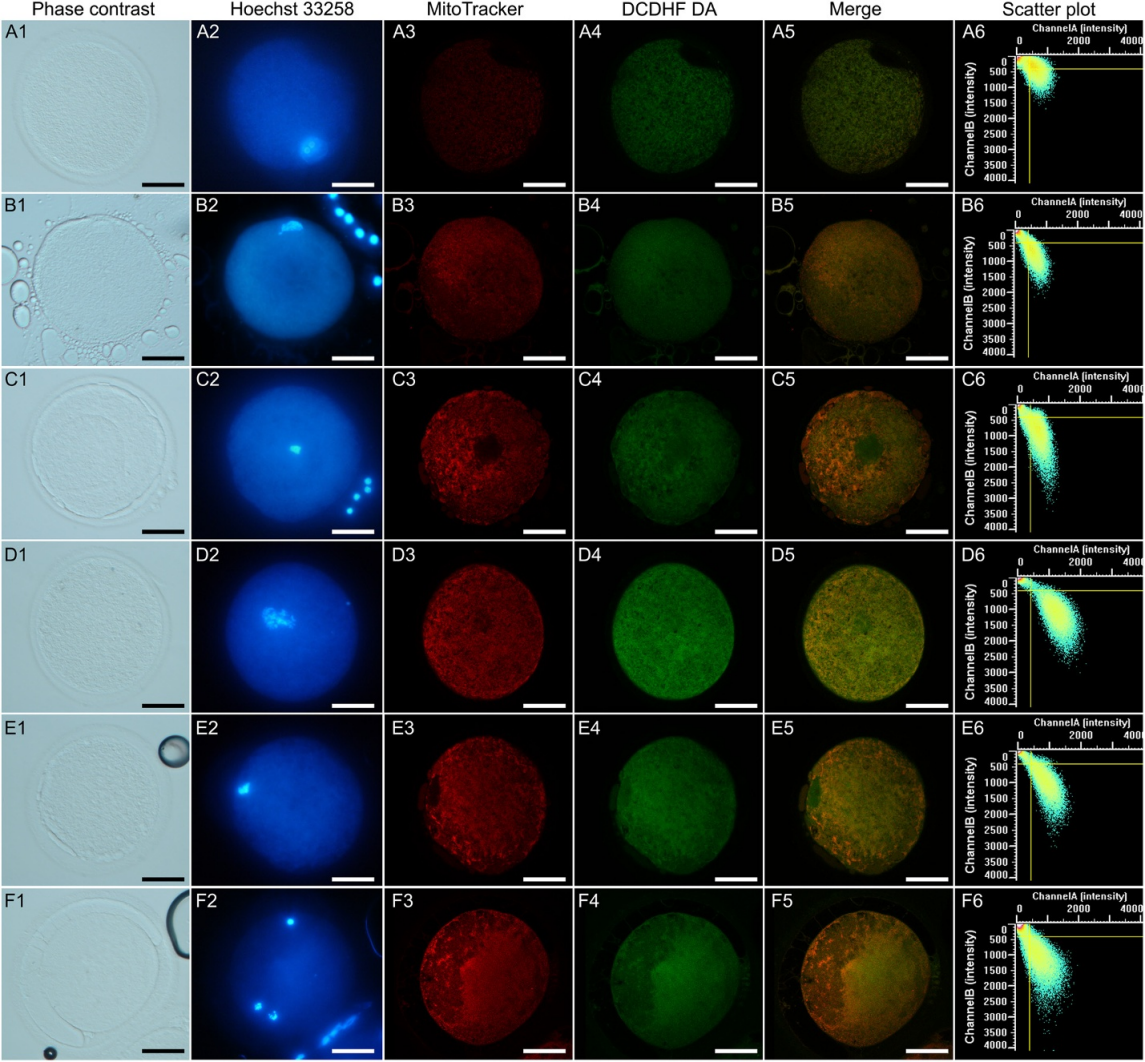
**Photomicrographs of equine oocytes fixed after recovery (IMM) or held overnight in EH medium at 25°C (EH), assessed for nuclear chromatin and energy/redox potential.** Bright field, epifluorescence and confocal images indicating morphology (A1-F1), chromatin configuration (A2-F2), mitochondrial (mt) distribution pattern (A3-F3), intracellular ROS localization (A4-F4), mt/ROS merged (A5-F5) and the mt/ROS colocalization scatter plot graph (A6-F6; Y axis: mt fluorescence intensity; X axis: ROS fluorescence intensity). **A**, EH-treated oocyte showing FN chromatin (A2), homogeneous distribution of small mt aggregates (SA; A3), ubiquitous ROS localization (A4), poor mt/ROS colocalization (A5), and low fluorescence intensity with wide red/green scatter, supporting lack of colocalization of ROS and mt signals (A6); **B**, EH-treated oocyte showing intermediate chromatin (B2), SA mt distribution (B3), but with higher mt intensity and greater mt/ROS colocalization compared to A (longer and narrower scatter plot); **C**, EH-treated oocyte showing condensed chromatin (C2; the nuclear outline in the mt image confirms this as a GV configuration), heterogenous perinuclear and pericortical mt pattern (P/P, C3), localized ROS distribution in areas with and without mt labeling (C4, C5), and the mt/ROS scatter plot (C6) showing greater fluorescence intensity than in A and B, with predominance of mt fluorescence (Y axis); **D**, IMM oocyte showing intermediate chromatin (D2), P/P mt pattern (D3), ROS localization in areas with and without mt labeling (D4, D5), and less precise mt/ROS colocalization, as shown by the broader scatter plot (D6); **E**, IMM oocyte showing condensed chromatin with nuclear outline and heterogeneous mt/ROS distribution, similar to D; **F**, IMM-MII oocyte, likely from an atretic follicle, showing MII chromatin at 7:00 (F2; cumulus cell nucleus at 12:00); P/P mt pattern (F3), ROS localization in areas with and without mt labeling (F4, F5), and a broad scatter plot, suggesting poor mt/ROS colocalization (F6). The scale bar represents 60 μm.

Quantitative data of fluorescence intensities for Mitotracker Orange CMTM Ros and DCF for 62 non-cultured oocytes are reported in Table 4. Two EH oocytes which had an abnormal mitochondrial distribution pattern, and an IMM CC oocyte with an abnormal shape were excluded from quantitative analysis. In non-cultured oocytes held in EH at controlled room temperature, there was no effect of EH treatment on mitochondrial activity, intracellular ROS levels, or Mitochondria/ROS colocalization at any meiotic stage. Within EH-treated oocytes, no differences were observed among chromatin configurations for any energy/redox parameter, however mitochondrial activity and intracellular ROS levels of oocytes showing FN chromatin configuration tended to be lower than those for the other configurations, as observed in Exp. 1. Within IMM oocytes, significantly higher mitochondrial activity and ROS levels were found in oocytes resuming meiosis compared with GV oocytes (F/I vs PI/MI and CC vs PI/MI; P <0.05). Photomicrographs representative of oocyte morphology, nuclear chromatin configuration, mitochondrial and ROS distribution patterns and Mitochondria/ROS colocalization of EH-25 and IMM oocytes are presented in Figure 4. The merged image shows the simultaneous distribution of overlapping fluorescence signals (corresponding to the Mitochondria- and ROS-specific probes) within the oocyte cytoplasmic area; an increasingly yellow image denotes increasing colocalization. The scatter plot displays how the two variables (MitoTracker and DCF fluorescent intensities) are correlated. Increasing width of the scatter denotes increasing variability, increasing length denotes increasing signal intensity. The chromatin categories with the highest prevalence are shown. Oocytes in lines A and B show homogeneous mitochondrial distribution and lower fluorescence intensity; oocytes in lines C-F show heterogeneous P/P mitochondrial pattern and higher fluorescence intensity, as also expressed by their longer scatter plot graphs. It should be noted that, in this figure, mitochondrial distribution are shown on only one confocal plane, whereas recording of distribution was performed using all 25 planes obtained.

**Table 4:** **Effects of holding in EH medium at controlled room temperature (25°C) on confocal energy/redox parameters of equine oocytes**

Treatment	Nuclear chromatin configuration	n. of analyzed oocytes	Mitochondrial activity	Intracellular ROS levels	Mitochondria/ROS colocalization
EH	FN	3	162.89 ± 101.19	172.93 ± 46.40	§
IMM		2	§	§	§
EH	F/I	4	437.35 ± 298.64	377.13 ± 181.20	0.40 ± 0.32
IMM		9	623.01 ± 376.96^a^	421.44 ± 164.87	0.38 ± 0.08
EH	CC	20	547.82 ± 499.54	278.53 ± 179.28	0.50 ± 0.10
IMM		19	722.93 ± 390.31^a^	377.92 ± 184.92	0.49 ± 0.19
EH	PI/MI	0	§	§	§
IMM		3	1292 ± 192.36^b^	643.67 ± 312.69	0.60 ± 0.10
EH	MII	0	§	§	§
IMM		2	§	§	§

Legend: Homogenous/heterogeneously fluorescent nucleus (*FN*); Fibrillar/Intermediate (*F/I*); Condensed chromatin (*CC*); Prometaphase I/Metaphase I (*PI/MI*); Metaphase II (*MII*). Mitochondrial activity and intracellular ROS levels are presented as MitoTracker and DCF fluorescence intensities expressed in Arbitrary Densitometric Units (*ADU*). Mitochondria/ROS colocalization is expressed as Pearson’s correlation coefficient. One-way ANOVA followed by Multiple Comparison Holm-Sidak method: ^a,b^ P <0.05; §: values for categories with fewer than 3 oocytes are not shown.

## Discussion

In the first study in this area, Torner et al. [39] presented the relationship between cumulus morphology and oocyte mitochondrial pattern in immature horse oocytes. The present study expands on these results to present the direct relationship of GV nuclear chromatin configuration to mitochondrial pattern and activity in equine immature oocytes. Torner et al. [39] reported significantly higher rates of heterogeneous mitochondrial pattern in oocytes at the diakinesis than at the diplotene stage. However, these authors had no category for the condensed chromatin configuration; they may have interpreted the CC configurations in GV oocytes as MI, as they report that 8 of 30 oocytes fixed at Time 0 were in MI. In our study, less than 5% of oocytes fixed directly had resumed meiosis. Torner et al. [39] matured oocytes for different times in vitro and reported increasing values of mitochondrial fluorescence intensities as oocytes progressed through meiosis, however, mitochondrial activity in immature oocytes showing different chromatin configurations within the GV was not reported. In a more recent paper, the same group reported mitochondrial pattern and activity of equine oocytes recovered by ultrasound-guided follicle aspiration, and fixed immediately [44]. In this study, no significant differences among chromatin configurations in mitochondrial activity or mitochondrial aggregation pattern was found. Thus, the present study is the first to demonstrate that in GV-stage equine oocytes, the CC configuration is associated with higher rates of the more mature mitochondrial distribution P/P and with higher mitochondrial activity, compared with the more juvenile FN and F/I stages.

The results of this study demonstrate that holding of oocytes overnight in a meiosis-inhibitor-free medium before maturation is not detrimental to oocyte mitochondrial status or redox status. Evaluation of the mitochondrial and oxidative status of immature oocytes was an effective measure of oocyte viability, as these findings were in agreement with the hypothesis that the FN configuration represents chromatin degeneration [9]. Of the IMM oocytes exhibiting the FN configuration, 44% showed an abnormal mitochondrial distribution pattern (Table 1). The mitochondrial activity in this group was higher than any other GV configuration, possibly reflecting generation of ROS during cell death. We noted a higher proportion of FN configuration in IMM oocytes in Experiment 1 than in Experiment 2; this may have been related to high environmental temperature during the period of this study. The more mature mitochondrial distribution found in immature oocytes having the CC configuration supports this configuration as representing the less juvenile, more meiotically-competent GV-stage oocyte, as previously reported [9, 45]. For CC oocytes, the mitochondrial distribution (Table 1) and activity, intracellular ROS levels, and Mitochondria/ROS colocalization (Table 2) values were remarkably similar between EH and IMM groups. This suggests that the CC configuration, which is also found in human oocytes in follicles of meiotically-competent size [46], is a stable germinal vesicle stage (persists unchanged during overnight holding). The maturing oocytes found in the IMM group were likely from atretic follicles [47], as oocytes in viable preovulatory equine follicles do not resume meiosis until beyond the maximum follicle size used for oocyte collection in this study.

Intermediate and CC chromatin configurations in GV-stage equine oocytes are competent to resume meiosis [9]. Thus, it could be predicted that the MII oocytes in the IMM group after IVM originated from both Intermediate and CC GV-stage oocytes, as these are present in about equal proportions in this group (Figure 1). However, the MII oocytes resulting in the EH group (Exp. 1) would appear to originate entirely from CC oocytes, as this was the only viable GV stage chromatin present after overnight holding (Figure 1). Therefore, the significant difference seen in mitochondrial distribution in IVM MII oocytes between the EH and IMM treatments (Table 1) may be due to the chromatin status of the GV oocytes at the time they were placed in maturation culture, with EH oocytes, having started with the more advanced CC chromatin, showing a higher prevalence of the P/P distribution, which is associated with cytoplasmic maturity [39–41].

Assessment of energy and oxidative capacity of EH oocytes in Exp. 1 provides qualitative and quantitative evidence that EH treatment does not lead to a state of respiratory cell stress, as this treatment was not detrimental to energy/redox parameters and in fact positively modified them (higher proportions of the P/P mitochondrial pattern). Mature (MII) oocytes from both IMM-IVM and EH-IVM treatments yielded significantly higher values for all energy/redox parameters when compared with those for immature oocytes, as reported previously [39].

The resumption of meiosis seen in non-cultured EH oocytes in Exp. 1 contradicted previous findings that EH holding effectively suppressed meiosis. Choi et al. [1] reported that equine oocytes in the EH treatment did not mature during holding (70% germinal vesicle stage after 18 h holding). Alm et al. [48] reported that the EH treatment maintained bovine oocytes at the germinal vesicle stage (79.3%, vs. 87.7% for control oocytes at 0 h; P >0.05). For these reasons, we performed a second study to examine the effects of temperature of holding on initial nuclear chromatin status. We found that holding at a consistent temperature of 25°C effectively maintained oocytes in the germinal vesicle stage, in agreement with previous studies (Figures 3 and 4), and showed for the first time that, at increased temperature, equine oocytes held in EH medium resume meiosis during holding; the low rate of normal MII oocytes found after IVM in the EH group in Experiment 1 suggests that increased temperature is detrimental to oocyte viability (Figure 3). Subsequently, we re-examined the effect of EH holding on oocyte Mitochondria/redox values. Holding in EH when performed at 25°C did not modify oocyte qualitative morphological (Mitochondria distribution pattern; Table 3) or quantitative (mitochondrial activity, intracellular ROS levels and Mitochondria/ROS colocalization; Table 4) energy/redox parameters. These are important findings given the increasing use of this procedure for both transport and for scheduling of maturation in equine oocytes for clinical and research uses.

## Conclusions

In summary, evaluation of energy/redox parameters was effective in defining progression of cytoplasmic maturation during EH treatment (uncontrolled temperature) and in detecting incompetent (FN) oocytes. EH holding does not compromise viability and energy/redox status in equine oocytes held at room temperature overnight, and progression of pre-meiotic chromatin configuration and mitochondrial status are dependent upon temperature. At variable room temperature, increased apparent cytoplasmic maturation before IVM was reflected in a more mature mitochondrial distribution in MII oocytes. Consistent holding at 25°C maintained oocytes in a state indistinguishable from IMM oocytes. Our findings support the use of EH pre-IVM treatment at 25°C to facilitate scheduling and oocyte transport. Further work is needed to determine if this may offer an option for laboratories working in farm animals as well as in human IVM. The absence of meiotic inhibitors in this medium provides a simple and potentially clinically-suitable pre-maturation method for application to assisted reproductive technologies.
